# Novel microbial modifications of bile acids and their functional implications

**DOI:** 10.1002/imt2.243

**Published:** 2024-10-13

**Authors:** Dan Zheng, Huiheng Zhang, Xiaojiao Zheng, Aihua Zhao, Wei Jia

**Affiliations:** ^1^ Center for Translational Medicine and Shanghai Key Laboratory of Diabetes Mellitus Shanghai Sixth People's Hospital Affiliated to Shanghai Jiao Tong University School of Medicine Shanghai China; ^2^ Department of Pharmacology and Pharmacy The University of Hong Kong Hong Kong China

## Abstract

This review outlines the recent discoveries of bile acids that have undergone novel microbial modifications, highlighting their biological roles and the profound implications for the development of innovative therapeutic strategies. The review aims to provide valuable insights and breakthroughs for future drug candidates in the expanding field of bile acid therapeutics.

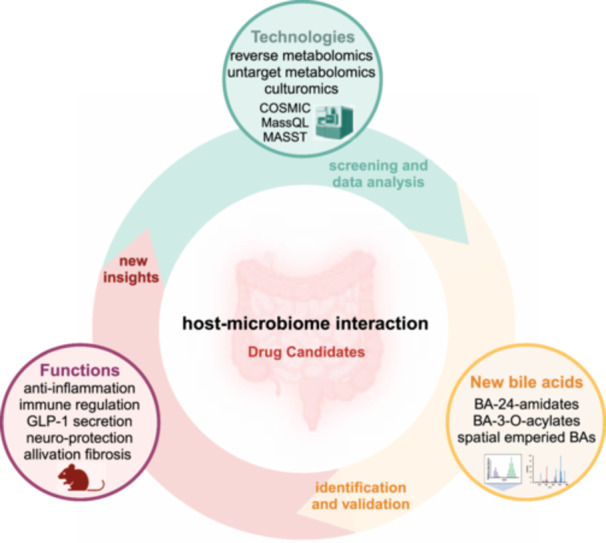

Bile acids (BAs) play a crucial role in facilitating fat digestion and absorption in the small intestine, as well as in regulating lipid and glucose metabolism, inflammation, and energy expenditure through signaling pathways [1]. These cholesterol‐derived biomolecules are synthesized in the liver and undergo four key modifications by the intestinal microbiota: deconjugation, 7α‐dehydroxylation, oxidation, and epimerization (Figure 1). Recent discoveries have identified reconjugated BAs as products of a fifth microbial modification, shedding new light on these processes and their impact on host physiology [2]. Significant progress has also been made in understanding the microbial enzymes responsible for 7α‐dehydroxylation and related stereoisomers. Moreover, the role of hyocholic acid (HCA) and hyodeoxycholic acid (HDCA) in metabolic diseases has garnered significant interest among researchers [3, 4, 5, 6, 7]. This review aims to consolidate the latest research findings on emerging BA molecules and evaluate their potential implications for the advancement of innovative therapeutic interventions, offering valuable insights to guide future drug innovation efforts.

**Figure 1:**
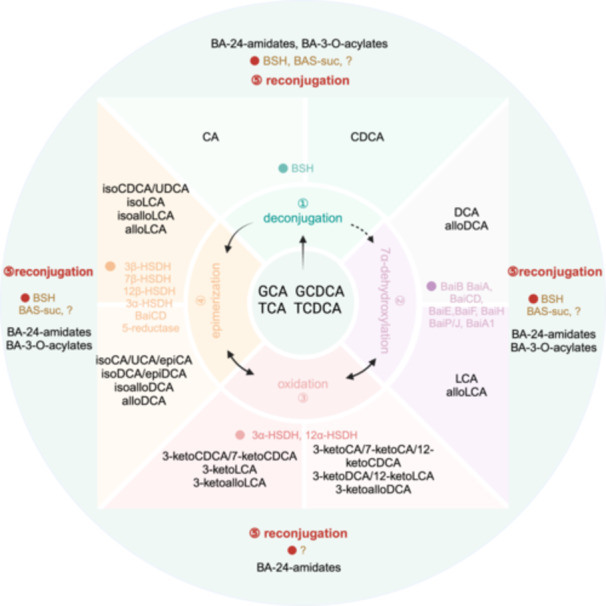
Gut microbial‐derived modifications of bile acids in the human intestinal tract. Gut microbes transform bile acids (BAs) through four primary pathways: deconjugation (removing glycine or taurine), 7α‐dehydroxylation, oxidation, and epimerization. Additionally, reconjugation as a fifth microbial modification involves the formation of BA‐24‐amidates through amidation of the C‐24 position of BAs with amino acids or polyamines, and the formation of BA‐3‐O‐acylates through esterification of the C‐3 position of BAs with fatty acids or organic acids. Black arrows indicate the transformation processes of BAs. Typically, conjugated cholic acid (CA)/chenodeoxycholic acid (CDCA) undergo deconjugation to form free CA/CDCA. CA/CDCA undergoes 7α‐dehydroxylation, primarily converting to deoxycholic acid (DCA)/lithocholic acid (LCA), with the production of by‐products alloDCA/alloLCA. This process also involves the formation of 3‐ketoDCA/3‐ketoLCA and isoDCA/isoLCA. DCA/LCA are further oxidized by enzymes produced by gut bacteria to form 3‐ketoDCA/3‐ketoLCA and epimerized to form isoDCA/isoLCA, epiDCA, as well as alloDCA/alloLCA. The epimerization process is bidirectional. Additionally, CA/DCA are also subjected to oxidation and epimerization catalyzed by gut bacteria. Green, purple, pink, yellow, and red circles represent the enzymes involved in the microbial metabolism of the aforementioned five types of bile acids that have been discovered to date. BSH, bile salt hydrolase; BaiB, bile acid CoA ligase; BaiA, 3α‐hydroxysteroid dehydrogenase; BaiCD, 3‐dehydro‐Δ^4^‐7α‐oxidoreductase; BaiE, 7α‐dehydratase; BaiF, CoA transferase; BaiH, 3‐dehydro‐Δ^4^‐7β‐oxidoreductase; BaiP/J, 5α‐reductase.

## GUT MICROBIAL‐DERIVED BA‐24‐AMIDATES AND THEIR BIOLOGICAL ROLES

Since Pieter C. Dorrestein's group identified three novel amino acid conjugations at the C‐24 position of cholic acid (CA) in 2020, which are modified by intestinal microbiota (Figure 1) [8], over 200 amino acid‐BA conjugates have been discovered to date (refer to Table S1).

Recent research has shown that various bacteria in the intestine, including species from the genera *Bacteroides*, *Lactobacillus*, *Bifidobacterium*, *Enterocloster*, *Ruminococcus*, and *Clostridium* are involved in catalyzing the conjugation of both primary and secondary BAs with amino acids. While bile salt hydrolase (BSH) is recognized for catalyzing the deconjugation of BA glyco‐ and tauro‐amidates, it also plays a role in the reamidation of BAs [8, 9]. Additionally, enzymes like those from *Clostridium scindens* ATCC 35704 can catalyze amino acid‐BA conjugation even without *bsh* gene [9]. Furthermore, the conjugation of BAs to glycine and taurine also occurs extrahepatically, a process previously thought to be liver‐specific [9].

Amino acid‐BA conjugates are highly abundant in the mouse cecum, cecal contents, colonic contents, and feces, which harbor a large number of gut microbiota. These conjugates are rarely detected in the liver, small intestine, and serum, likely due to their low basal levels of production in vivo [2]. Nonetheless, amino acid‐BA conjugates can enter the enterohepatic circulation intact, alanoCA, aspartylCA, glutamidoCA, leucoCA, phenylalanoCA, seroCA, threonoCA, and tyrosoCA were observed throughout the entire gastrointestinal tract, as well as in the liver, kidney, serum, and gallbladder [9]. High levels of phenylalanoCA and seroCA were found across all tissues and organs [9].

**Figure 2:**
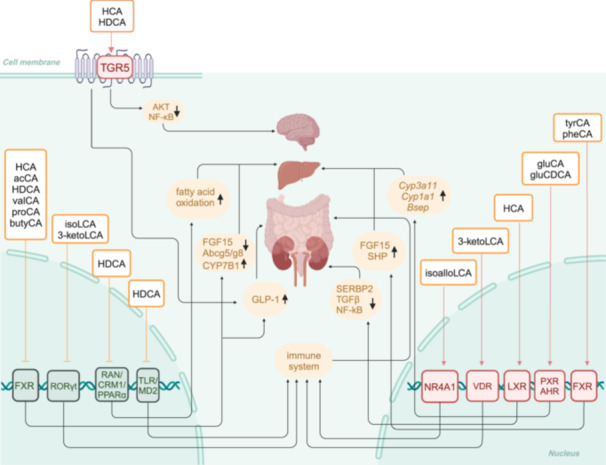
Role of gut microbial‐derived bile acids on receptor regulation. The 3‐O‐acyl‐cholic acids (3‐O‐acyl‐CAs), 3‐acetylCA (acCA), 3‐propionylCA (proCA), 3‐butyrylcholic acid (butyCA), and 3‐valerylCA (valCA) are antagonists of FXR (farnesoid X receptor). PhenylalanoCA (pheCA) and tyrosoCA (tyrCA) activate the FXR and regulate bile acid (BA) biosynthesis genes. GlutamidoCA (gluCA) and glutamidoCDCA (gluCDCA) activate pregnane X receptor (PXR) and aryl hydrocarbon receptor (AHR) and upregulated cholesterol 3 alpha‐hydroxylase 1a (*Cyp3a1*), cholesterol 1 alpha‐hydroxylase 1 (*Cyp1a1*) and bile salt export protein (*Bsep*) expression. 3‐Ketolithocholica acid (3‐ketoLCA) and isolithocholic acid (isoLCA) inhibit retinoid‐related orphan receptor‐γt (RORγt), which suppresses T helper 17 (T_H_17) cell differentiation. IsoalloLCA binds to nuclear receptor 4A1 (NR4A1), upregulating the expression of forkhead box protein 3 (Foxp3), which in turn promotes the differentiation of T regulatory (T_reg_) cells. 3‐ketoLCA activates the vitamin D receptor (VDR), modulating the function of RORγt^+^ T_reg_ cells. Hyocholic acid (HCA) and hyodeoxycholic acid (HDCA) inhibit FXR signaling, promoting the production and secretion of glucagon‐like peptide‐1 (GLP‐1) in the intestine. HDCA inhibits FXR, decreases Fgf15 expression, increases the expression CYP7B1, and decreases the expression of the cholesterol transporter Abcg5/g8 gene in the liver. HDCA disrupting the ras‐related nuclear protein/chromosomal region maintenance 1/peroxisome proliferator‐activated receptor alpha (RAN/CRM1/PPARα) heterotrimer and enhancing PPARα's nuclear presence, thereby promoting fatty acid oxidation and curbing inflammation. HCA activates the renal liver X receptor (LXR), which in turn downregulates the expression of sterol regulatory element‐binding proteins2 (SREBP2), transforming growth factor‐β (TGF‐β), and nuclear factor κB (NF‐κB). HCA and HDCA activate the G protein‐coupled BA receptor (TGR5), promoting the production and secretion of glucagon‐like peptide‐1 (GLP‐1) in the intestine. Moreover, HDCA activates TGR5, which in turn downregulates the activity of the protein kinase B (AKT) and NF‐κB pathways in brain. HDCA interacts directly with the toll‐like receptor 4/myeloid differentiation factor 2 receptor (TLR4/MD2) complex, competitively inhibiting the binding of lipopolysaccharide (LPS) to TLR4/MD2. The light red border indicates that the receptor is activated, while the dark green color signifies that the receptor is inhibited.

Fecal phenylalanoCA, tyrosoCA, and leuco/ileucoCA, have been found to be increased in Crohn's disease patients but not in those with ulcerative colitis, potentially providing a means to distinguish and diagnose the specific type of inflammatory bowel disease (IBD) [2]. Moreover, amino acid‐BA conjugates may promote intestinal inflammation by modulating the production of IFNγ in T cells and activating the pregnane X receptor (PXR) [10]. PhenylalanoCA and tyrosoCA, can activate the human farnesoid X receptor (FXR) and increase the expression of FXR‐target genes, which are crucial for BA synthesis in the liver [2]. Chenodeoxycholic acid (CDCA) conjugates are the majority of activators of the human BA receptor FXR, which can also activate the transcription PXR and aryl hydrocarbon receptor (AHR) (Figure 2) [8].

Polyamine‐BA conjugates are another type of BA‐24‐amidates [11]. Agmatine, putrescine, N‐acetyl‐putrescine, N‐carbamoyl‐putrescine, spermidine, N‐acetyl‐spermidine, N‐acetyl‐spermine, cadaverine, N‐acetyl‐cadaverine, and 1,3‐diaminopropane, can form as polyamine‐BA conjugates, as recently discovered by Pieter C. Dorrestein's group [11]. The levels of polyamine conjugates vary with Mediterranean or typical American diet styles [11].

## GUT MICROBIAL‐DERIVED BA‐3‐O‐ACYLATES AND THEIR BIOLOGICAL ROLES

Recent studies have identified BA‐3‐O‐acylates of CA, CDCA, and major secondary BAs such as deoxycholic acid (DCA), lithocholic acid (LCA), isoDCA, and isoLCA, conjugated with short‐chain fatty acids (SCFAs) ranging from C1:0 to C5:0 and long‐chain fatty acids (LCFAs), mainly C16:0 and C18:0, in both human and mouse feces (refer to Table S2) [1, 12]. Additionally, BAs have been esterified with binary carboxylic acids (refer to Table S2) in healthy individuals and mice [13]. Two valine conjugates were identified as esters rather than amidates when CDCA and 3‐ketoCDCA were cultured with gut microbiota (refer to Table S2) [14]. *Bacteroides* has been identified as a key player in BA esterification in gut microbiota [1]. In vitro studies have shown that 11 bacterial species have the ability to catalyze the binding of SCFAs and CA, with *Christensenella* species capable of transforming all SCFA‐CA conjugates [12]. Jiang's group discovered BAS‐suc, a β‐lactamase responsible for the esterification of succinic acid and CA in *Bacteroides uniformis* [13].

BA‐3‐O‐acylates exhibit an intestine‐specific distribution. This specificity is due to the degradation of certain components in the duodenal contents, which hinders their absorption into the bloodstream [12]. Further research is needed to understand the detailed mechanisms involved in their biodistribution.

Studies have shown that 3‐O‐acyl‐CAs, including 3‐acetyl‐, 3‐propionyl‐, 3‐butyryl‐, and 3‐valerylCA, are depleted in individuals with type 2 diabetes (T2D), suggesting their potential in improving T2D outcomes [12]. The administration of 3‐succinylCA on metabolic dysfunction‐associated steatohepatitis (MASH) mice was efficacious in alleviating symptoms by promoting the growth of *Akkermansia muciniphila* [13], suggesting its potential as a small molecular therapeutic agent. While BA‐3‐O‐acylates represent a promising avenue for the treatment of metabolic diseases, further research is needed to fully understand their mechanisms and potential therapeutic applications.

## THE ENZYMES AND IMMUNOBIOLOGICAL ROLES OF GUT MICROBIAL‐DERIVED 7α‐DEHYDROXYLATION AND EPIMERIZATION

The process of 7α‐dehydroxylation has long been recognized as a fundamental microbial biotransformation. However, the specific pathway of 7α‐dehydroxylation of BAs, also known as the Hylemon‐Björkhem pathway, was only elucidated in 2020 [1, 15]. This pathway involves stereoisomerization of the A/B rings, as well as 3‐oxidation and 3‐epimerization, leading to the production of alloBAs, isoalloBAs, 3‐ketoBAs, and isoBAs. This diversification of BA structures is driven by a series of enzymatic reactions (Figure 1, Table S3) [1].

The epimers of DCA and LCA, isoDCA and isoLCA, can also be formed by a two‐step process involving epimerization at the C‐3 hydroxyl group of DCA and LCA. It begins with the oxidation of the C‐3 hydroxyl group by 3α‐hydroxysteroid dehydrogenase (3α‐HSDH), followed by reduction by 3β‐HSDH to yield isoDCA and isoLCA. A variety of intestinal bacteria from Actinobacteria and Firmicutes possess the enzyme 3α‐HSDH. *Gordonibacter pamelaeae*, *Eggerthella lenta*, *Clostridium citroniae*, and *Ruminococcuse gnavus* possess 3β‐HSDH. In a similar way, DCA is converted to 12‐ketoLCA by 12α‐HSDH. Subsequently, the enzyme 12β‐HSDH catalyzes the conversion of 12‐ketoLCA to epiDCA [1].

Recent research has revealed that isoalloLCA and 3‐ketoLCA promote T regulatory (T_reg_) cells differentiation while hindering T helper 17 (T_H_17) cells differentiation. The levels of isoalloLCA, isoLCA in patient samples with IBD, and their biosynthetic genes are generally lower than those in the control group [1, 16].

Additionally, isoDCA can reduce dendritic cell inflammation and promote T_reg_ cell production, helping to maintain intestinal immune homeostasis. The use of isoDCA and isoLCA may aid IBD management by promoting T_reg_ cell differentiation and inhibiting T_H_17 cell development. Iso‐, 3‐keto‐, allo‐, 3‐ketoallo‐, and isoalloLCA have been found enriched in Japanese centenarians, and isoalloLCA is responsible for against gram‐positive multidrug‐resistant pathogens, which is involved in the risk of pathogen infection [1].

## HCA'S ROLES IN METABOLIC DISEASES

There has been growing interest in the bioactivities and mechanisms of HCA species BAs (Figure 2), including HCA, HDCA, and their glycine and taurine conjugates, which are rare BAs in humans, and whose biosynthesis and metabolism are not yet fully understood.

Our group's previous studies have identified HCA species as predictive biomarkers and a promising multi‐target therapeutic agent for T2D [3]. HCA species activate the G protein‐coupled BA receptor (TGR5) and inhibit FXR signaling, promoting the production and secretion of glucagon‐like peptide‐1 (GLP‐1) and effectively regulating blood glucose homeostasis [4]. Additionally, our study also found that patients with metabolic dysfunction‐associated fatty liver disease (MAFLD) exhibit decreased serum levels of HCA species, particularly HDCA and glycoHDCA [6]. HDCA stimulates hepatic alternative BA synthetic pathway by inhibiting intestinal FXR, and suppress the hepatic classical BA synthetic pathway by modulation of gut microbiota to activate peroxisome proliferator activated receptor alpha (PPARα) signaling pathway [6]. Moreover, HDCA binds to ras‐related nuclear protein within hepatocytes, boosting PPARα levels and enhancing fatty acid oxidation, thus reducing inflammation and improving MAFLD [7].

Additionally, HDCA has been shown to have several other beneficial effects, including inhibiting the formation of cholesterol gallstones, attenuating inflammation and protecting against sepsis, and preventing central neuroinflammation [17, 18, 19]. HCA has been found to reduce renal cholesterol synthesis and accumulation, ameliorate high‐energy diet‐induced kidney fibrosis, and improve lipid metabolism disorders and immune responses [20].

## METABOLOMIC TECHNIQUES FOR DISCOVERING NOVEL BAS

The discovery of novel BA molecules relies on advanced metabolomic techniques (refer to Table S4). Combining unbiased untargeted metabolic profiling with public databases searches, such as the Global Natural Products Social Molecular Networking system (GNPS) and BAFinder, enables researcher to identify potential BA derivatives with distinct core backbone. Targeted metabolomics quantify the known BAs, and its effectiveness in identifying novel BA constituents may be constrained by the reliance on existing knowledge and the availability of reference standards.

Reverse metabolomics is a novel technique for discovering novel metabolites. When bio‐samples are screened using untargeted metabolomics, all metabolite features are compared with those in public databases and the newly synthesized metabolites to unveil potential metabolites associated with specific phenotypes, species, and sample types [10]. Retention time‐based annotation strategy and ion mobility spectrometry are effectively used to address isomers and epimers in BA amidates. Additionally, the combination of culturomics with metabolomics has proven to be a successful approach for discovering novel BAs in biological samples [12, 13, 14]. Molecular network construction tools such as Mass Spectrometry Search Tool, Mass Spec Query Language, Confidence Of Small Molecule IdentifiCations, and other mass data analysis methods are essential for the discovery of novel BAs.

## PERSPECTIVE FOR FUTURE BA RESEARCH AND NEW DRUG DISCOVERY

In the past 5 years, over 250 novel microbially modified BAs have been discovered through a combination of mass spectrometry technologies, publicly available data resources, and bioinformatic computational tools. With the increasing application of artificial intelligence (AI) technology in the biomedical field, we anticipate that AI can facilitate the screening of vast amounts of previously unknown types of BAs and the exploration of the specific microbiota involved in BA modification. AI can also predict the function of enzymes involved in BA metabolism, unveil new modification pathways, or even reconstruct the known microbial metabolic pathways of BAs.

Moreover, considering both bacterial and fungal modification of BAs, exploring fungal modification of BAs may be a promising frontier for future research in this field, we can gain a more comprehensive understanding of the complex interplay between the gut microbiota and host health.

BAs serve as natural endogenous ligands, forming a solid foundation for the development of semi‐synthetic derivatives with enhanced properties, positioning them as leading compounds in drug candidate screening. For instance, INT‐787 is currently undergoing phase II clinical trials for severe alcohol‐associated hepatitis. EDP‐305, a side chain derivative of obeticholic acid, is a promising FXR agonist being evaluated for its potential in treating MASH. Aramchol, a stearoyl CoA desaturase 1 inhibitor developed by Galmed Pharmaceuticals, is in phase III clinical trials for MASH and liver fibrosis. HTD1801 (also known as berberine ursodeoxycholate) is undergoing clinical trials for multiple indications, including MASH, T2D, severe hypertriglyceridemia, primary sclerosing cholangitis, and primary biliary cholangitis.

From these clinical trials, we can infer that microbial modification of the BA backbone, can enhance or change the affinity of BA derivatives to their receptors in the host, resulting in new bioactivities. Importantly, this provides lead compounds for discovering new drug candidates in the future. Subsequent studies are expected to reveal additional variations in these modifications under different health conditions, thereby enhancing our comprehension of their multifaceted roles in bodily functions.

## CONCLUSION

BA research has unveiled the intricate interplay between the host and the microbiome, shedding light on how this interaction profoundly influences host metabolism. Leveraging advanced analytical techniques and metabolomics technologies, researchers can expedite the exploration of previously undiscovered microbial modifications of BAs and efficiently screen potential new drug candidates.

## AUTHOR CONTRIBUTIONS

**Dan Zheng**: Writing—review and editing; writing—original draft; data curation; visualization; funding acquisition. **Huiheng Zhang**: Data curation. **Xiaojiao Zheng**: Writing—review and editing; funding acquisition. **Aihua Zhao**: Writing—original draft; writing—review and editing; funding acquisition; data curation; visualization. **Wei Jia**: Supervision; funding acquisition; conceptualization; writing—original draft; writing—review and editing; project administration; visualization.

## CONFLICT OF INTEREST STATEMENT

The authors declare no conflict of interest.

## ETHICS STATEMENT

No animals or humans were involved in this study.

## Supporting information


**Table S1.** Bacterial taxa and enzyme contributing to catalyzing bile acid 24‐amidates.
**Table S2.** Bacterial taxa and enzyme contributing to catalyzing bile acid‐3‐O‐acylates.
**Table S3.** Bacterial taxa and enzyme contributing to catalyzing DCA and LCA derivatives.
**Table S4.** Strategy for discovering novel conjugated novel bile acids.

## Data Availability

No new data and scripts were used for this review. Supplementary materials (tables, graphical abstract, slides, videos, Chinese translated version and update materials) may be found in the online DOI or iMeta Science http://www.imeta.science/. Data sharing is not applicable to this article as no new data were created or analyzed in this study.
