# Supplementary material for: Novel microbial modifications of bile acids and their functional implications
Source: Imeta. 2024 Oct 13;3(5):e243. doi: 10.1002/imt2.243 (PMC11487544; doi:10.1002/imt2.243)
Supplement: Supplementary file 1 — Table S1. Bacterial taxa and enzyme contributing to catalyzing bile acid 24‐amidates. Table S2. Bacterial taxa and enzyme contributing to catalyzing bile acid‐3‐O‐acylates. Table S3. Bacterial taxa and enzyme contributing to catalyzing DCA and LCA derivatives. Table S4. Strategy for discovering novel conjugated novel bile acids. [file IMT2-3-e243-s001.docx]

**Supporting Information to**

**Novel Microbial Modifications of Bile Acids and Their Functional** **Implications**

**Running title**: Microbial-derived bile acids and their function

Dan Zheng^1^, Huiheng Zhang^1^, Xiaojiao Zheng^1^, Aihua Zhao^1*^, Wei Jia^1,2*^

^1^Center for Translational Medicine and Shanghai Key Laboratory of Diabetes Mellitus, Shanghai Sixth People’s Hospital Affiliated to Shanghai Jiao Tong University School of Medicine, Shanghai 200233, China

^2^Department of Pharmacology and Pharmacy, University of Hong Kong, Hong Kong, China

*Correspondence: weijia2@hku.hk (Wei Jia), zhah@sjtu.edu.cn (Aihua Zhao)

**Supplementary Tables**

**Table S1** Bacterial taxa and enzyme contributing to catalyze bile acid 24-amidates

**Table S2** Bacterial taxa and enzyme contributing to catalyze bile acid-3-O-acylates

**Table S3** Bacterial taxa and enzyme contributing to catalyze DCA and LCA derivatives

**Table S4** Strategy for discovering novel conjugated novel bile acids

**Table S1**. Bacterial taxa and enzyme contributing to catalyze bile acid 24-amidates

| **Bile acid 24-amidates** | **Bacteria** | **Enzymes** | **Refs.** |
| --- | --- | --- | --- |
| **proteinogenic amino acid conjugates**^a^ | | | |
| alanoCA, β-alanoCA, alanoCDCA, alano-α-MCA, β-alano-α-MCA, alano-β-MCA, β-alano-β-MCA, β-alano-7-ketoCA, β-alano-12-ketoDCA, alanoDCA, β-alanoDCA, alanoLCA, alano-ω-MCA, β-alano-ω-MCA, alanoHDCA, β-alanoHDCA, alano-isoCA, alanoUCA, alano-epiisoCA, alanoUDCA, alano-isoDCA, alano-epiDCA | Genus: *Clostridium*, *Ruminococcus*; Species: *Bacteroides fragilis*, *Bacteroides ovatus*, *Bacteroides uniformis*, *Bacteroides xylanisolvens*, *Bifidobacterium adolescentis*, *Bifidobacterium bifidum*, *Bifidobacterium dentium*, *Bifidobacterium longum*, *Bifidobacterium pseudocatenulatum*, *Blautia luti*, *Blautia producta*, *Clostridium perfringens*, *Clostridium scindens*, *Collinsella aerofaciens*, *Coprococcus comes*, *Dorea formicigenerans*, *Dorea longicatena*, *Enterocloster bolteae*, *Enterocloster clostridioformis*, *Enterococcus faecalis*, *Holdemania filiformis*, *Hungatella hathewayi*, *Lactiplantibacillus plantarum*, *Ruminococcus gnavus*, *Streptococcus infantarius*, *Tyzzerella nexilis* | BSH | [1-8] |
| arginoCA, arginoCDCA, argino-β-MCA, arginoDCA, arginoLCA, argino-ω-MCA, arginoHDCA | Genus: *Clostridium*, *Ruminococcus.* Species: *Coprococcus comes*, *Holdemania filiformis*, *Lactobacillus ruminis*, *Roseburia intestinalis*, *Ruminococcus gnavus*, *Streptococcus infantarius*, *Tyzzerella nexilis* | BSH | [1, 3, 5, 6, 8] |
| asparaginylCA, asparaginylCDCA, asparaginyl-α-MCA, asparaginyl-β-MCA, asparaginylDCA, asparaginylLCA, asparaginyl-ω-MCA | Genus: *Clostridium*, *Ruminococcus*; *S*pecies: *Bifidobacterium bifidum*, *Bifidobacterium longum*, *Blautia producta*, *Clostridium perfringens*, *Clostridium symbiosum*, *Coprococcus comes*, *Dorea formicigenerans*, *Enterocloster bolteae*, *Enterococcus faecalis*, *Lactiplantibacillus plantarum*, *Ruminococcus gnavus*, *Streptococcus infantarius* | BSH | [1, 5, 6, 8] |
| aspartylCA, aspartylCDCA, aspartylDCA, aspartyl-ω-MCA | Genus: *Clostridium*, *Ruminococcus*; Species: *Bifidobacterium bifidum*, *Blautia producta*, *Clostridium perfringens*, *Clostridium scindens*, *Enterocloster aldenensis*, *Enterocloster bolteae*, *Enterocloster citroniae*, *Enterococcus faecalis*, *Holdemania filiformis*, *Hungatella hathewayi*, *Lacrimispora celerecrescens*, *Lactiplantibacillus plantarum*, *Ruminococcus gnavus* |  | [1, 8, 9] |
| cysteoCA | *Bifidobacterium bifidum*, *Blautia producta*, *Clostridium perfringens*, *Clostridium scindens*, *Enterocloster bolteae*, *Enterocloster clostridioformis*, *Enterococcus faecalis*, *Hungatella hathewayi*, *Lactiplantibacillus plantarum*, *Ruminococcus gnavus* | BSH | [5] |
| glutamylCA, glutamylCDCA, glutamyl-β-MCA, glutamylDCA, glutamylMDCA, glutamylLCA, glutamylHCA, glutamylHDCA, glutamylACA, glutamylUDCA, glutamyl-isoDCA, glutamyl-epiDCA, glutamyl-isoLCA | *Bifidobacterium bifidum*, *Bifidobacterium longum*, *Bifidobacterium pseudocatenulatum*, *Blautia producta*, *Coprococcus comes*, *Enterocloster bolteae*, *Enterococcus faecalis*, *Lactiplantibacillus plantarum*, *Ruminococcus gnavus*, *Streptococcus infantarius* | BSH | [1, 5-8] |
| glutamidoCA, glutamidoCDCA, D-glutamidoCDCA, glutamido-α-MCA, glutamido-β-MCA, glutamidoDCA, D-glutamidoDCA, glutamidoLCA, glutamido-ω-MCA, glutamidoHDCA, glutamido-isoCA, glutamidoUDCA, glutamido-isoDCA, glutamido-epiDCA | Genus: *Clostridium*, *Ruminococcus*; Species: *Bacteroides caccae*, *Bacteroides ovatus*, *Bacteroides uniformis*, *Bacteroides xylanisolvens*, *Bifidobacterium adolescentis*, *Bifidobacterium bifidum*, *Bifidobacterium dentium*, *Bifidobacterium longum*, *Bifidobacterium pseudocatenulatum*, *Blautia producta*, *Clostridium perfringens*, *Clostridium scindens*, *Coprococcus comes*, *Dorea longicatena*, *Enterocloster bolteae*, *Enterococcus faecalis*, *Lactiplantibacillus plantarum*, *Roseburia intestinalis*, *Ruminococcus gnavus*, *Streptococcus infantarius*, *Tyzzerella nexilis* | BSH | [1, 5-8] |
| histidoCA, histidoCDCA, histido-β-MCA, histidoDCA, histidoLCA, histido-isoDCA, histido-epiDCA, histido-isoLCA | Genus: *Clostridium*, *Ruminococcus*; Species: *Bacteroides fragilis*, *Bifidobacterium pseudocatenulatum*, *Clostridium perfringens*, *Clostridium symbiosum*, *Coprococcus comes*, *Enterocloster bolteae*, *Enterococcus faecalis*, *Holdemania filiformis*, *Lactiplantibacillus plantarum*, *Lactobacillus ruminis*, *Ruminococcus gnavus*, *Streptococcus infantarius* | BSH | [1, 5-7] |
| leuco/isoleucoCA, leuco/isoleucoCDCA, leuco/isoleuco-α-MCA, leuco/isoleuco-β-MCA, leuco/isoleuco-diketoCDCA, ile-7ketoHCA, leuco/isoleuco-diketoHDCA, leuco/isoleuco-DCA, leuco/isoleucoLCA, leu-ω-MCA, leuco/isoleucoHDCA, leuco/isoleuco-UDCA, leuco/isoleuco-isoDCA, leuco/isoleuco-epiDCA, leuco/isoleuco-isoLCA, leuco/isoleuco-isoalloLCA | Genus: *Clostridium*, *Ruminococcus*; Species: *Bacteroides ovatus*, *Bacteroides thetaiotaomicron*, *Bacteroides xylanisolvens*, *Bifidobacterium adolescentis*, *Bifidobacterium bifidum*, *Blautia producta*, *Clostridium perfringens*, *Clostridium scindens*, *Collinsella aerofaciens*, *Coprococcus comes*, *Enterocloster bolteae*, *Enterocloster clostridioformis*, *Enterococcus faecalis*, *Holdemania filiformis*, *Hungatella hathewayi*, *Lactiplantibacillus plantarum*, *Lactobacillus ruminis*, *Roseburia intestinalis*, *Ruminococcus gnavus*, *Streptococcus infantarius* | BSH | [1-4, 6-8, 10] |
| lysoCA, lysoCDCA, lyso-α-MCA, lyso-β-MCA, lysoDCA, N1-lyso-DCA, N6-lyso-DCA, lys-ω-MCA, lys-isoDCA, N1-lyso-isoDCA, N6-lyso-isoDCA, N1-lyso-epiDCA, N6-lyso-epiDCA, lys-12epiDCA, N1-lyso-isoLCA, N6-lyso-isoLCA | *Blautia producta*, *Clostridium perfringens*, *Clostridium symbiosum*, *Enterocloster bolteae*, *Enterocloster clostridioformis*, *Enterocloster lavalensis*, *Enterococcus faecalis*, *Holdemania filiformis*, *Lactiplantibacillus plantarum*, *Lactobacillus ruminis*, *Ruminococcus gnavus* | BSH | [1, 3, 5-8] |
| methionoCA, methionoCDCA, methiono-α-MCA, methiono-β-MCA, methionoDCA, methionoUDCA | Genus: *Clostridium*, *Ruminococcus*; Species: *Bacteroides caccae*, *Bacteroides xylanisolvens*, *Bifidobacterium adolescentis*, *Bifidobacterium bifidum*, *Bifidobacterium dentium*, *Bifidobacterium pseudocatenulatum*, *Clostridium scindens*, *Coprococcus comes*, *Dorea formicigenerans*, *Dorea longicatena*, *Enterocloster bolteae*, *Lactobacillus ruminis*, *Roseburia intestinalis*, *Ruminococcus gnavus*, *Streptococcus infantarius*, *Tyzzerella nexilis* | BSH | [1, 5, 6] |
| phenylalanoCA, phenylalanoCDCA, phenylalano-α-MCA, phenylalano-β-MCA, phenylalano-3-ketoCA, phenylalano-12-ketoCA, phenylalano-3-ketoCDCA, phenylalano-diketoCDCA, phenylalano-12-ketoLCA, phenylalano-diketoHDCA, phenylalanoDCA, phenylalanoLCA, phenylalano-ω-MCA, phenylalanoHDCA, phenylalano-isoCA, phenylalano-apoCA, phenylalanoUDCA, phenylalano-isoDCA, phenylalano-epiDCA, phenylalano-12-epiDCA | Genus: *Clostridium*, *Ruminococcus*; Species: *Bacteroides caccae*, *Bacteroides fragilis*, *Bacteroides ovatus*, *Bacteroides thetaiotaomicron*, *Bacteroides xylanisolvens*, *Bifidobacterium adolescentis*, *Bifidobacterium bifidum*, *Bifidobacterium dentium*, *Bifidobacterium longum*, *Bifidobacterium pseudocatenulatum*, *Blautia luti*, *Blautia producta*, *Clostridium bolteae*, *Clostridium perfringens*, *Clostridium scindens*, *Collinsella aerofaciens*, *Coprococcus comes*, *Dorea longicatena*, *Enterocloster bolteae*, *Enterococcus faecalis*, *Holdemania filiformis*, *Hungatella hathewayi*, *Lactiplantibacillus plantarum*, *Lactobacillus ruminis*, *Roseburia intestinalis*, *Ruminococcus gnavus*, *Streptococcus infantarius*, *Tyzzerella nexilis* | BSH | [1, 2, 4-8, 10] |
| proloCA, prolo-α-MCA, prolo-β-MCA, proloDCA, proloLCA |  |  | [2, 3, 6] |
| seroCA, sero-β-MCA, seroDCA | *Bacteroides fragilis*, *Bacteroides ovatus*, *Bacteroides xylanisolvens*, *Bifidobacterium adolescentis*, *Bifidobacterium bifidum*, *Bifidobacterium dentium*, *Bifidobacterium longum*, *Bifidobacterium pseudocatenulatum*, *Blautia producta*, *Clostridium perfringens*, *Coprococcus comes*, *Dorea longicatena*, *Enterocloster bolteae*, *Enterococcus faecalis*, *Lacrimispora indolis*, *Lacrimispora sphenoides*, *Lactiplantibacillus plantarum*, *Ruminococcus gnavus*, *Streptococcus infantarius*, *Tyzzerella nexilis* | BSH | [1, 3-6] |
| threonoCA, threonoCDCA, threono-α-MCA, threonoDCA, threono-ω-MCA | *Bacteroides fragilis*, *Bifidobacterium bifidum*, *Bifidobacterium longum*, *Blautia producta*, *Clostridium perfringens*, *Enterocloster bolteae*, *Enterococcus faecalis*, *Lacrimispora indolis*, *Lacrimispora sphenoides*, *Lactiplantibacillus plantarum*, *Ruminococcus gnavus* | BSH | [3-9] |
| tryptoCA, tryptoCDCA, trypto-α-MCA, trypto-β-MCA, trypto-diketoCDCA, tryptoDCA, tryptoLCA, trypto-ω-MCA, tryptoHCA, tryptoHDCA, tryptoUDCA, trypto-isoDCA, trypto-epiDCA, trypto-isoLCA | Genus: *Clostridium*, *Ruminococcus*; Species: *Blautia producta*, *Clostridium perfringens*, *Coprococcus comes*, *Enterocloster bolteae*, *Enterococcus faecalis*, *Lactiplantibacillus plantarum*, *Lactobacillus ruminis*, *Roseburia intestinalis*, *Ruminococcus gnavus*, *Streptococcus infantarius* | BSH | [1, 3, 5-8] |
| tyrosoCA, tyrosoCDCA, tyroso-α-MCA, tyroso-β-MCA, tyroso-3-ketoCA, tyroso-12-ketoCA, tyroso-diketoCDCA, tyroso-diketoHDCA, tyrosoDCA, tyrosoHDCA, tyroso-ω-MCA, tyrosoUDCA, tyroso-isoDCA, tyroso-epiDCA, tyroso-isoLCA | Genus: *Clostridium*, *Ruminococcus*; Species: *Bacteroides fragilis*, *Bacteroides thetaiotaomicron*, *Bacteroides xylanisolvens*, *Bifidobacterium adolescentis*, *Bifidobacterium bifidum*, *Blautia luti*, *Blautia producta*, *Clostridium bolteae*, *Clostridium perfringens*, *Coprococcus comes*, *Dorea longicatena*, *Enterocloster bolteae*, *Enterococcus faecalis*, *Holdemania filiformis*, *Lactiplantibacillus plantarum*, *Lactobacillus ruminis*, *Roseburia intestinalis*, *Ruminococcus gnavus*, *Streptococcus infantarius*, *Tyzzerella nexilis* | BSH | [1, 4-7, 10] |
| valoCA, valoCDCA, valo-α-MCA, valo-β-MCA, valo-7-ketoLCA, valoDCA, valoMDCA, valoLCA, valo-ω-MCA, valoHDCA, valo-epiisoCA, valoUCA, valo-isoDCA, valo-epiDCA, valo-isoLCA | *Coprococcus comes*, *Holdemania filiformis*, *Hungatella hathewayi*, *Lactobacillus ruminis*, *Streptococcus infantarius* | BSH | [1, 3, 5-8] |
| **non-proteinogenic amino acid conjugates^a^** | | | |
| γ-aminobutyrylCA, γ-aminobutyrylCDCA, γ-aminobutyrylDCA | *Bacteroides fragilis*, *Bacteroides ovatus*, *Bifidobacterium longum*, *Mediterraneibacter gnavus* | Ntn | [11] |
| 5-aminovalerylCA, 5-aminovalerylCDCA |  |  | [12] |
| citrulloCA, citrulloCDCA, citrulloDCA | *Bifidobacterium bifidum*, *Blautia producta*, *Clostridium perfringens*, *Enterocloster bolteae*, *Enterococcus faecalis*, *Ruminococcus gnavus* |  | [5, 9, 13] |
| 2-aminoisobutyrylCA, 2-aminoisobutyryl-12-ketoDCA, 2-aminoisobutyrylDCA, 2-aminoisobutyryl-ω-MCA |  |  | [8] |
| ornithoCA, ornitho-α-MCA, ornitho-β-MCA, ornithoDCA, ornitho-ω-MCA |  |  | [8, 13] |
| tyramoylCA, tyramoylDCA | *Bacteroides fragilis*, *Bacteroides ovatus*, *Bifidobacterium longum*, *Mediterraneibacter gnavus* | Ntn | [11] |
| alanyl-alanoLCA, alanyl-alanoDCA, alanyl-alanoHDCA |  |  | [8] |
| lysyl(iso-)-glycoCA, lysyl(iso-)-glycoCDCA |  |  | [8] |
| **polyamine conjugates**^b^ | | | |
| cholyl-1,3-diaminopropane |  |  | [12] |
| cholyl-agmatine |  |  | [12] |
| cholyl-cadaverine |  |  | [12] |
| cholyl-putrescine, chenodeoxycholyl-putrescine |  |  | [12] |
| cholyl-spermidine |  |  | [12] |
| cholyl-N-acetyl-cadaverine, chenodeoxycholyl-N-acetyl-cadaverine |  |  | [12] |
| cholyl-N-acetyl-putrescine, chenodeoxycholyl-N-acetyl-putrescine |  |  | [12] |
| cholyl-N-acetyl-spermidine, chenodeoxycholyl-N-acetyl-spermidine |  |  | [12] |
| cholyl-N-acetyl-spermine |  |  | [12] |
| cholyl-N-carbamoyl-putrescine |  |  | [12] |

^a^ In the reported literatures, there are various naming conventions for amino acid amidates, and this article uniformly adopts one of them.

^b^ The substances are named according to the reference 12.

CA, cholic acid; CDCA, chenodeoxycholic acid; DCA, deoxycholic acid; LCA, lithocholic acid; UDCA, ursodeoxycholic acid; MCA, muricholic acid; HCA, hycocholic acid; HDCA, hycodeoxycholic acid.

**Table S2**. Bacterial taxa and enzyme contributing to catalyze bile acid-3-O-acylates

| Bile acid 3-O-acylates | Bacteria | Enzymes | Refs. |
| --- | --- | --- | --- |
| short fatty acid conjugates^a^ | | | |
| 3-formylCA |  |  | [14] |
| 3-acetylCA, 3-acetylCDCA, 3-acetylDCA, 3-acetylLCA, 3-acetylUDCA, 3-acetylHDCA, 3-acetyl-isoDCA, 3-acetyl-isoLCA, 3-acetyl-isoUDCA/MDCA | *Bacteroides difficilis*; *Christensenella hongkongensis*, *Christensenella minuta*, *Christensenella tenuis*; *Gehongia tenuis*; *Blautia segnis*; *Parabacteroides distasonis*, *Parabacteroides hominis*; *Phocaeicola dorei* |  | [14-16] |
|  |  |  |  |
| 3-propionylCA | *Christensenella hongkongensis*, *Christensenella minuta*, *Christensenella tenuis* |  | [14, 16] |
| 3-butyrylCA | *Christensenella hongkongensis*, *Christensenella minuta*, *Christensenella tenuis* |  | [14, 16] |
| 3-valerylCA | *Christensenella hongkongensis*, *Christensenella minuta*, *Christensenella tenuis*, *Lawsonibacter faecis* |  | [16] |
| long fatty acid conjugates^a^ | | | |
| 3-myristylLCA |  |  | [13] |
| 3-palmityl-isoDCA, 3-palmityl-isoLCA |  |  | [15, 17] |
| 3-stearylCA, 3-stearyl-isoDCA, 3-stearyl-isoLCA |  |  | [13, 15, 17] |
| 3-oleyl-isoDCA, 3-oleyl-isoLCA |  |  | [15, 17] |
| 3-linoleyl-isoDCA, 3-linoleyl-isoLCA, 3-linoleyl-CA |  |  | [13, 15] |
| 3-octadecenoylCA |  |  | [13] |
| 3-linolenylCA |  |  | [13] |
| 3-arachidonylCA |  |  | [13] |
| 3-docosahexaenoylCDCA |  |  | [13] |
| organic acid conjugates | | | |
| 3-glycolylCA |  |  | [14] |
| 3-malylCA |  |  | [14] |
| 3-succinylCA (3-succinylated CA) | *Bacillus aerius*; *Bacteroides fragilis*, *Bacteroides uniformis*; *Bifidobacterium longum*; *Citrobacter freundii*; *Clostridium tertium*; *Eggerthella lenta*; *Enterococcus avium*, *Enterococcus durans*, *Enterococcus faecalis*, *Enterococcus faecium*; *Faecalicoccus pleomorphus*; *Fusobacterium nucleatum*; *Lacrimispora xylanolytica*; *Parabacteroides goldsteinii*; *Phocaeicola dorei*; *Ruminococcus gnavus*; *Shigella flexneri*; *Shigella sonnei* | BAS-suc | [14] |
| amino acid conjugates^b^ |  |  |  |
| valolithocholate ester |  |  | [5] |
| leucolithocholate ester |  |  | [5] |

^a^ In the reported literatures, there are various naming conventions for fatty acid acylates, and this article uniformly adopts one of them.

^b^ The substances are named according to the reference 3.

CA, cholic acid; CDCA, chenodeoxycholic acid; DCA, deoxycholic acid; LCA, lithocholic acid.

**Table S3** Bacterial taxa and enzyme contributing to catalyze DCA and LCA derivatives

| **Bile acids** | **Precursors** | **Bacteria** | **Enzymes** | **Refs.** |
| --- | --- | --- | --- | --- |
| **allo-bile acids** | | | | |
| alloDCA | CA, DCA | Genus: *Eubacterium*; Species: *Clostridium scindens*, *Eggerthella lenta* | BaiB, BaiA, BaiCD, BaiE, BaiF, BaiH, BaiP/J, BaiA1;  3α-HSDH, BaiCD,  5-reductase | [18, 19] |
| alloLCA | CDCA, LCA | *Clostridium scindens* | BaiB, BaiA, BaiCD, BaiE, BaiF, BaiH, BaiP/J, BaiA1;  3α-HSDH, BAiCD,  5-reductase | [18] |
| **iso-bile acids and 3-keto bile acids** | | | | |
| isoDCA | DCA | Family:Lachnospiraceae; Species: *Eggerthella lenta*, *Ruminococcus gnavus* | 3α-HSDH, 3β-HSDH | [19-21] |
| isoLCA | LCA, isoUDCA | Family:Lachnospiraceae, Odoribacteraceae, Ruminococcaceae, Dorea; Species: *Bacillus coagulans*, *Bacteroides cellulosilyticus*, *Bacteroides dorei*, *Bacteroides fragilis*, *Bacteroides rodentium*, *Bacteroides thetaiotaomicron*, *Bacteroides uniformis*, *Bacteroides vulgatus*, *Bifidobacterium pseudocatenulatum*, *Catenibacterium mitsuokai*, *Clostridium aldenense*, *Clostridium innocuum*, *Collinsella aerofaciens*, *Eggerthella lenta*, *Emergencia timonensis*, *Faecalicatena contorta*, *Gordonibacter pamelaeae*, *Hungatella hathewayi*, *Lachnospira pectinoschiza*, *Lactobacillus rogosae*, *Parabacteroides distasonis*, *Parabacteroides merdae*, *Peptoniphilus harei*, *Ruminococcus gnavus* | 3α-HSDH, 3β-HSDH; BaiN | [19-22] |
| 3-ketoDCA | CA, DCA, isoDCA | *Clostridium hiranonis*, *Clostridium hylemonae*, *Clostridium scindens*, *Ruminococcus gnavus* | BaiB, BaiA, BaiCD, BaiE, BaiF, BaiH, BaiP/J; 3α-HSDH | [19, 20, 23-25] |
| 3-ketoLCA | CDCA, LCA, isoLCA | Family: Lachnospiraceae, Odoribacteraceae, Christensenellaceae, Desulfovibrionaceae, Ruminococcaceae; Species: *Adlercreutzia equolifaciens*, *Akkermansia muciniphila*, *Bacteroides thetaiotaomicron*, *Bifidobacterium longum*, *Clostridiales*, *Clostridium aldenense*, *Clostridium citroniae*, *Clostridium hylemonae*, *Clostridium innocuum*, *Clostridium perfringens*, *Clostridium scindens*, *Clostridium symbiosum*, *Collinsella aerofaciens*, *Collinsella intestinalis*, *Corynebacterium striatum*, *Eggerthella lenta*, *Emergencia timonensis*, *Faecalicatena contorta*, *Gordonibacter pamelaeae*, *Hungatella hathewayi*, *Monoglobus pectinilyticus*, *Parabacteroides distasonis*, *Parabacteroides merdae*, *Peptoniphilus harei*, *Phascolarctobacterium faecium*, *Phocea massiliensis*, *Pyramidobacter piscolens*, *Raoultibacter massiliensis*, *Ruminococcus gnavus* | BaiB, BaiA, BaiCD, BaiE, BaiF, BaiH, BaiP/J; 3α-HSDH | [19, 20, 23] |
| isoalloLCA | alloLCA | Family:Odoribacteraceae; Species: *Alistipes finegoldii*, *Alistipes indistinctus*, *Alistipes onderdonkii*, *Bacillus coagulans*, *Bacteroides caccae*, *Bacteroides cellulosilyticus*, *Bacteroides dorei*, *Bacteroides eggerthii*, *Bacteroides finegoldii*, *Bacteroides fragilis*, *Bacteroides ovatus*, *Bacteroides rodentium*, *Bacteroides thetaiotaomicron*, *Bacteroides uniformis*, *Bacteroides vulgatus*, *Bifidobacterium pseudocatenulatum*, *Butyricimonas synergistica*, *Catenibacterium mitsuokai*, *Clostridium scindens*, *Collinsella aerofaciens*, *Eggerthella lenta*, *Lachnospira pectinoschiza*, *Lactobacillus rogosae*, *Odoribacter laneus*, *Parabacteroides chongii*, *Parabacteroides goldsteinii*, *Parabacteroides merdae*, *Peptoniphilus harei*, *Ruminococcus gnavus* | 3α-HSDH, 3β-HSDH | [26, 27] |
| isoalloDCA | alloDCA | Family:Odoribacteraceae; Species: *Alistipes finegoldii*, *Alistipes indistinctus*, *Alistipes onderdonkii*, *Bacillus coagulans*, *Bacteroides caccae*, *Bacteroides cellulosilyticus*, *Bacteroides dorei*, *Bacteroides eggerthii*, *Bacteroides finegoldii*, *Bacteroides fragilis*, *Bacteroides ovatus*, *Bacteroides rodentium*, *Bacteroides thetaiotaomicron*, *Bacteroides uniformis*, *Bacteroides vulgatus*, *Bifidobacterium pseudocatenulatum*, *Butyricimonas synergistica*, *Catenibacterium mitsuokai*, *Clostridium scindens*, *Collinsella aerofaciens*, *Eggerthella lenta*, *Lachnospira pectinoschiza*, *Lactobacillus rogosae*, *Odoribacter laneus*, *Parabacteroides chongii*, *Parabacteroides goldsteinii*, *Parabacteroides merdae*, *Peptoniphilus harei*, *Ruminococcus gnavus* | 3α-HSDH, 3β-HSDH | [26, 27] |
| 3-keto-alloDCA | CA, alloDCA, isoalloDCA | Genus: *Eubacterium*; Species: *Clostridium scindens* | BaiB, BaiA, BaiCD, BaiE, BaiF, BaiH, BaiP/J; 3α-HSDH, 3β-HSDH | [18, 24] |
| 3-keto-alloLCA | CDCA, alloLCA, isoalloLCA | *Clostridium scindens* | BaiB, BaiA, BaiCD, BaiE, BaiF, BaiH, BaiP/J; 3α-HSDH, 3β-HSDH | [18] |
| **epi-bile acid and 12-keto bile acid** | | | | |
| epiDCA | CA, DCA | *Clostridium paraputrificum*, *Clostridium scindens*, *Clostridium tertium* | 12α-HSDH,  12β-HSDH | [28, 29] |
| 12-ketoLCA | DCA, epiDCA | Genus: *Clostridium*; *Eggerthella*; Species: *Egibacterium lentum*, *Saccharopolyspora rectivirgula* | 12α-HSDH,  12β-HSDH | [29-31] |

CA, cholic acid; CDCA, chenodeoxycholic acid; DCA, deoxycholic acid; LCA, lithocholic acid; isoUDCA, ursodeoxycholic acid.

**Table S4** Strategy for discovering novel conjugated novel bile acids

| **Strategy** | **Characteristics** | **Notes** | **Refs.** |
| --- | --- | --- | --- |
| Untargeted metabolomics | Comprehensive analysis of metabolites in biological specimen. | Limited by the sensitivity of instrument and availability of database. | [8, 10, 12] |
| Targeted metabolomics | Detecting and quantifying target substances in biological samples. | Limited by corresponding reference standards. Typically, this method only analyzes the targeted known metabolites, which may overlook other unknown or untargeted known metabolites in the sample. | [6, 7] |
| Reverse metabolomics | MS spectra acquired from newly synthesized compounds are searched for in public metabolomics datasets to uncover phenotypic associations. | Obtaining MS/MS spectra is fundamental for identifying metabolites in the context of reverse metabolomics, with the technique most effective for compounds that can be chemically synthesized. However, there are limitations in its ability to differentiate isomers due to challenges in resolving structurally similar compounds. | [13] |
| Culturomics combined with metabolomics | Providing optimal conditions for the co-culture of gut bacteria and small molecules in vitro to enhance the generation of microbial-derived metabolites, followed by their analysis using targeted or untargeted analytical methods | Due to the limitations of culturing intestinal bacteria in vitro, some modifications and transformations that may exist in vivo are missed. | [3, 14, 16] |

**References**

1. Lucas, L. N., K. Barrett, R. L. Kerby, Q. Zhang, L. E. Cattaneo, D. Stevenson, F. E. Rey, D. Amador-Noguez. 2021. “Dominant Bacterial Phyla from the human gut show widespread ability to transform and conjugate bile acids.” *mSystems* e0080521. <https://doi.org/10.1128/mSystems.00805-21>

2. Zhu, Quan-Fei, Yan-Zhen Wang, Na An, Jun-Di Hao, Peng-Cheng Mei, Ya-Li Bai, Yu-Ning Hu, Pei-Rong Bai, Yu-Qi Feng. 2022. “Alternating dual-collision energy scanning mass spectrometry approach: discovery of novel microbial bile-acid conjugates.” *Analytical Chemistry* 94: 2655-2664. <https://doi.org/10.1021/acs.analchem.1c05272>

3. Garcia, Carlos J., Vit Kosek, David Beltrán, Francisco A. Tomás-Barberán, Jana Hajslova. 2022. “Production of new microbially conjugated bile acids by human gut microbiota.” *Biomolecules* 12: 687. <https://doi.org/10.3390/biom12050687>

4. Rimal, Bipin, Stephanie L. Collins, Ceylan E. Tanes, Edson R. Rocha, Megan A. Granda, Sumeet Solanki, Nushrat J. Hoque, et al. 2024. “Bile salt hydrolase catalyses formation of amine-conjugated bile acids.” *Nature* 626: 859-863. <https://doi.org/10.1038/s41586-023-06990-w>

5. Guzior, Douglas V., Maxwell Okros, Madison Shivel, Bruin Armwald, Christopher Bridges, Yousi Fu, Christian Martin, et al. 2024. “Bile salt hydrolase acyltransferase activity expands bile acid diversity.” *Nature* 626: 852-858. <https://doi.org/10.1038/s41586-024-07017-8>

6. Zhang, Yang, Yuxi Huang, Jingjing Fan, Meng Zhang, Aobulikasimu Hasan, Yang Yi, Rong Yu, Xujie Zhou, Min Ye, Xue Qiao. 2022. “Expanding the scope of targeted metabolomics by one-pot microscale synthesis and tailored metabolite profiling: investigation of bile acid–amino acid conjugates.” *Analytical Chemistry* 94: 16596-16603. <https://doi.org/10.1021/acs.analchem.2c02086>

7. Wang, Yan-Zhen, Peng-Cheng Mei, Pei-Rong Bai, Na An, Jin-Gang He, Jie Wang, Quan-Fei Zhu, Yu-Qi Feng. 2023. “A strategy for screening and identification of new amino acid-conjugated bile acids with high coverage by liquid chromatography-mass spectrometry.” *Analytica Chimica Acta* 1239: 340691. <https://doi.org/10.1016/j.aca.2022.340691>

8. Ma, Yan, Yang Cao, Xiaocui Song, Weichen Xu, Zichen Luo, Jinjun Shan, Jingjie Zhou. 2023. “Integration of semi-empirical MS/MS library with characteristic features for the annotation of novel amino acid-conjugated bile acids.” *The Analyst* 148: 5380-5389. <https://doi.org/10.1039/d3an01237a>

9. Stewart, Allison K., Matthew H. Foley, Michael K. Dougherty, Sarah K. McGill, Ajay S. Gulati, Emily C. Gentry, Lee R. Hagey, et al. 2023. “Using multidimensional separations to distinguish isomeric amino acid–bile acid conjugates and assess their presence and perturbations in model systems.” *Analytical Chemistry* 95: 15357-15366. <https://doi.org/10.1021/acs.analchem.3c03057>

10. Quinn, Robert A., Alexey V. Melnik, Alison Vrbanac, Ting Fu, Kathryn A. Patras, Mitchell P. Christy, Zsolt Bodai, et al. 2020. “Global chemical effects of the microbiome include new bile-acid conjugations.” *Nature* 579: 123-129. <https://doi.org/10.1038/s41586-020-2047-9>

11. Mullowney, M. W., A. Fiebig, M. K. Schnizlein, M. McMillin, A. R. Rose, J. Koval, D. Rubin, et al. 2024. “Microbially catalyzed conjugation of GABA and tyramine to bile acids.” *Journal of Bacteriology logo* 206: e0042623. <https://doi.org/10.1128/jb.00426-23>

12. Mohanty, Ipsita, Helena Mannochio-Russo, Joshua V. Schweer, Yasin El Abiead, Wout Bittremieux, Shipei Xing, Robin Schmid, et al. 2024. “The underappreciated diversity of bile acid modifications.” *Cell* 187: 1801-1818. <https://doi.org/10.1016/j.cell.2024.02.019>

13. Gentry, Emily C., Stephanie L. Collins, Morgan Panitchpakdi, Pedro Belda-Ferre, Allison K. Stewart, Marvic Carrillo Terrazas, Hsueh-han Lu, et al. 2023. “Reverse metabolomics for the discovery of chemical structures from humans.” *Nature* 626: 419-426. <https://doi.org/10.1038/s41586-023-06906-8>

14. Nie, Qixing, Xi Luo, Kai Wang, Yong Ding, Shumi Jia, Qixiang Zhao, Meng Li, et al. 2024. “Gut symbionts alleviate MASH through a secondary bile acid biosynthetic pathway.” *Cell* 187: 2717-2734. <https://doi.org/10.1016/j.cell.2024.03.034>

15. Takei, Hajime, Seiko Narushima, Mitsuyoshi Suzuki, Genta Kakiyama, Takahiro Sasaki, Tsuyoshi Murai, Yuichiro Yamashiro, Hiroshi Nittono. 2022. “Characterization of long-chain fatty acid-linked bile acids: a major conjugation form of 3β-hydroxy bile acids in feces.” *Journal of Lipid Research* 63: 100275. <https://doi.org/10.1016/j.jlr.2022.100275>

16. Liu, Chang, Meng-Xuan Du, Li-Sheng Xie, Wen-Zhao Wang, Bao-Song Chen, Chu-Yu Yun, Xin-Wei Sun, et al. 2024. “Gut commensal *Christensenella minuta* modulates host metabolism via acylated secondary bile acids.” *Nature Microbiology* 9: 434-450. <https://doi.org/10.1038/s41564-023-01570-0>

17. Kelsey, M. I., J. E. Molina, S. K. Huang, K. K. Hwang. 1980. “The identification of microbial metabolites of sulfolithocholic acid.” *Journal of Lipid Research* 21: 751-759. <https://doi.org/10.1016/s0022-2275(20)34802-1>

18. Lee, J. W., E. S. Cowley, P. G. Wolf, H. L. Doden, T. Murai, K. Y. O. Caicedo, L. K. Ly, et al. 2022. “Formation of secondary allo-bile acids by novel enzymes from gut Firmicutes.” *Gut Microbes* 14: 2132903. <https://doi.org/10.1080/19490976.2022.2132903>

19. Devlin, A. S., M. A. Fischbach. 2015. “A biosynthetic pathway for a prominent class of microbiota-derived bile acids.” *Nature Chemical Biology* 11: 685-690. <https://doi.org/10.1038/nchembio.1864>

20. Campbell, Clarissa, Peter T. McKenney, Daniel Konstantinovsky, Olga I. Isaeva, Michail Schizas, Jacob Verter, Cheryl Mai, et al. 2020. “Bacterial metabolism of bile acids promotes generation of peripheral regulatory T cells.” *Nature* 581: 475-479. <https://doi.org/10.1038/s41586-020-2193-0>

21. Bai, Y., T. Zhao, M. Gao, Y. Zou, X. Lei. 2022. “A novel gene alignment in Dorea sp. AM58-8 produces 7-dehydroxy-3β bile acids from primary bile acids.” *Biochemistry* 61: 2870-2878. <https://doi.org/10.1021/acs.biochem.2c00264>

22. Zhang, Boyan, Xianzhe Jiang, Yue Yu, Yimeng Cui, Wei Wang, Hailing Luo, Sokratis Stergiadis, Bing Wang. 2024. “Rumen microbiome-driven insight into bile acid metabolism and host metabolic regulation.” *The ISME Journal* 18: <https://doi.org/10.1093/ismejo/wrae098>

23. Funabashi, Masanori, Tyler L. Grove, Min Wang, Yug Varma, Molly E. McFadden, Laura C. Brown, Chunjun Guo, Steven Higginbottom, Steven C. Almo, Michael A. Fischbach. 2020. “A metabolic pathway for bile acid dehydroxylation by the gut microbiome.” *Nature* 582: 566-570. <https://doi.org/10.1038/s41586-020-2396-4>

24. Ridlon, J. M., D. J. Kang, P. B. Hylemon. 2010. “Isolation and characterization of a bile acid inducible 7alpha-dehydroxylating operon in *Clostridium hylemonae* TN271.” *Anaerobe* 16: 137-146. <https://doi.org/10.1016/j.anaerobe.2009.05.004>

25. Doden, H., L. A. Sallam, S. Devendran, L. Ly, G. Doden, S. L. Daniel, J. M. P. Alves, J. M. Ridlon. 2018. “Metabolism of oxo-bile acids and characterization of recombinant 12α-Hydroxysteroid dehydrogenases from bile acid 7α-dehydroxylating human gut bacteria.” *Applied and Environmental Microbiology* 84: e00235-00218. <https://doi.org/10.1128/aem.00235-18>

26. Li, Wei, Saiyu Hang, Yuan Fang, Sena Bae, Yancong Zhang, Minghao Zhang, Gang Wang, et al. 2021. “A bacterial bile acid metabolite modulates Treg activity through the nuclear hormone receptor NR4A1.” *Cell Host & Microbe* 29: 1366-1377. <https://doi.org/10.1016/j.chom.2021.07.013>

27. Sato, Yuko, Koji Atarashi, Damian R. Plichta, Yasumichi Arai, Satoshi Sasajima, Sean M. Kearney, Wataru Suda, et al. 2021. “Novel bile acid biosynthetic pathways are enriched in the microbiome of centenarians.” *Nature* 599: 458-464. <https://doi.org/10.1038/s41586-021-03832-5>

28. Doden, Heidi L., Patricia G. Wolf, H. Rex Gaskins, Karthik Anantharaman, João M. P. Alves, Jason M. Ridlon. 2021. “Completion of the gut microbial epi-bile acid pathway.” *Gut Microbes* 13: 1907271. <https://doi.org/10.1080/19490976.2021.1907271>

29. Edenharder, R., J. Schneider. 1985. “12 beta-Dehydrogenation of bile acids by *Clostridium paraputrificum*, *C. tertium*, and *C. difficile* and epimerization at carbon-12 of deoxycholic acid by cocultivation with 12 alpha-dehydrogenating *Eubacterium lentum*.” *Applied and Environmental Microbiology* 49: 964-968. <https://doi.org/10.1128/aem.49.4.964-968.1985>

30. Mythen, S. M., S. Devendran, C. Méndez-García, I. Cann, J. M. Ridlon. 2018. “Targeted synthesis and characterization of a gene cluster encoding NAD(P)H-dependent 3α-, 3β-, and 12α-hydroxysteroid dehydrogenases from *Eggerthella CAG*:298, a gut metagenomic sequence.” *Applied and Environmental Microbiology* 84: e02475-02417. <https://doi.org/10.1128/aem.02475-17>

31. Kollerov, V. V., D. Monti, N. O. Deshcherevskaya, T. G. Lobastova, E. E. Ferrandi, A. Larovere, S. A. Gulevskaya, S. Riva, M. V. Donova. 2013. “Hydroxylation of lithocholic acid by selected actinobacteria and filamentous fungi.” *Steroids* 78: 370-378. <https://doi.org/10.1016/j.steroids.2012.12.010>
